# Association of salivary levels of immunoglobulin-a and amylase with oral-dental manifestations in patients with controlled and non-controlled type 2 diabetes

**DOI:** 10.1186/s12903-019-0868-4

**Published:** 2019-08-06

**Authors:** Marjan Kheirmand Parizi, Hamed Akbari, Mahsa Malek-mohamadi, Maryam Kheirmand Parizi, Shahla Kakoei

**Affiliations:** 1 Dentist, Student Research Committee, School of Dentistry, Kerman University of Medical Sciences, Kerman, Iran; 2 Endocrinology and Metabolism Research Center, Institute of Basic and Clinical Physiology Sciences, Kerman University of Medical Sciences, Kerman, Iran; 3 Department of Biochemistry, School of Medicine, Kerman University of Medical Sciences, Kerman, Iran; 4 Community Oral Health Department, School of dentistry, Shahid Beheshti University of Medical Sciences, Tehran, Iran; 5 Student Research Committee, Shahid Beheshti University of Medical Sciences, Tehran, Iran; 6 Oral and Dental Diseases Research Center, Dental School, Kerman University of Medical Sciences, Kerman, Iran; 7 Department of Oral Medicine, School of Dentistry, Kerman University of Medical Sciences, Kerman, Iran

**Keywords:** Diabetes mellitus, Saliva, Salivary immunoglobulin-a, Salivary amylase, Oral manifestations

## Abstract

**Background:**

Oral health is related to general health and one of the most prevalent chronic diseases is diabetes mellitus. Diabetes can have adverse effects on oral health and vice versa. Saliva analysis can be used as a non-invasive method to obtain information about diseases status like diabetes. The aim of present study was to evaluate the salivary immunoglobulin-A (s-IgA) and salivary amylase levels and their associations with oral-dental manifestations in patients with controlled and non-controlled type 2 diabetes.

**Methods:**

This case-control study was carried out on 90 subjects who referred to the Diabetes Center of Shahid Bahonar Hospital, Kerman University of Medical Sciences, Kerman, Iran. Participants were divided into three groups: 1) uncontrolled diabetic patients (*n* = 30); 2) controlled diabetic patients (*n* = 30); and 3) healthy individuals (*n* = 30). Unstimulated salivary levels of I-A and amylase were measured. All participants underwent a dental and periodontal examination to explore the oral and dental manifestations. T-test, chi-square and ANOVA tests were used for data analysis in SPSS 18.

**Results:**

Significant higher level of s-IgA was found in uncontrolled diabetic patients compared to controlled diabetic (*P* ≤ 0.0001) and the control group (*P* = 0.004). Moreover, the mean levels of s-amylase in uncontrolled patients was significantly higher compared to controlled diabetic (*P* = 0.01) and the control group (P ≤ 0.0001). Uncontrolled diabetic patients with oral candidiasis, erythematous candidiasis, abscesses, or xerostomia had higher s-IgA levels compared to the controlled diabetic participants. Moreover, uncontrolled diabetic patients with oral candidiasis or erythematous candidiasis showed a significant higher levels of s-amylase compared to controlled diabetic patients. Also, significant positive correlations were found between s-IgA and DMFT and s-IgA and PDI (r = 0.444, *P* = 0.014 and r = 0.386, *P* = 0.035, respectively).

**Conclusion:**

In conclusion, higher s-amylase and s-IgA concentrations may reflect oral-dental manifestations in T2DM. Moreover, the current findings suggest that s-amylase and s-IgA may serve as a complementary and alternative fluid in screening for diabetes mellitus.

## Background

Type 2 diabetes mellitus (T2DM) is a chronic and progressive disease threatening people throughout the world, particularly in developing countries [1, 2]. Currently, T2DM is characterized by the World Health Organization (WHO) as the sixth leading cause of death globally, and it is estimated that 439 million adults will be affected by T2DM by the year 2030 [3]. Regardless of its prevalence, the hormonal changes, microvascular, macrovascular, and neuronal injuries associated with T2DM cause complications such as retinopathy, nephropathy, neuropathy, and oral-dental manifestations [4, 5]. It has been well established that T2DM is associated with some oral cavities complications affecting the quality of life [6]. The most prevalent oral/dental complications include xerostomia, burning mouth syndrome, dental caries, tooth loss, periapical lesions, various infections, oral candidiasis, lichen planus, odontogenic abscess, taste disturbance, salivary glands dysfunction and specially periodontal diseases such as gingivitis and periodontitis [7, 8].

Accumulating evidence indicates that saliva is a non-invasive source for the detection of different oral and systemic conditions and is an essential fluid for protecting the dynamic environment of the oral cavity [9, 10]. Some studies have shown that the permeability of basement membranes of the salivary glands are altered in diabetic patients [11–13]. Submandibular, parotid, and sublingual salivary glands are exocrine glands assigned to secreting saliva. The main components of salivary fluid are water, electrolytes, and proteins. Salivary proteins such as enzymes (amylase, lipase), albumin, immunoglobulin, glycoproteins, polypeptides, and oligopeptides play a critical role in oral health [9, 14].

Secretory IgA (s-IgA) is an antimicrobial defense agent against pathogens in the first line of the immune system, and it plays a major role in oral health [15]. It is noteworthy that previous investigations have merely evaluated IgA concentrations in uncontrolled diabetic patients; however, these studies have yielded conflicting results [16–20].

Amylase is a calcium-dependent enzyme found in saliva that breaks starch down into maltose and dextrin [21]. The greater penetration of proteins such as amylase in saliva due to changes in the base membrane permeability of the salivary glands is seen in diabetic patients, and some studies have shown a higher expression of amylase receptors in diabetes [22]. Considering these findings, the current study evaluated salivary amylase levels in controlled and uncontrolled diabetic patients. The results of studies by Kim [16] and by Prathibha [17] showed lower levels of amylase in diabetic objects, while the previous findings [18–20] reported higher amounts of s-amylase in the participants. Some other studies have declared no changes in s-amylase amounts in diabetic patients [13, 23].

The aims of the current study were first, to evaluate salivary IgA and amylase levels and their associations with diabetes; second, to compare the results of subjects with oral/dental manifestations among the controlled T2DM, uncontrolled T2DM, and healthy participants; and finally, to reveal the correlations between variables.

## Methods

This case-control retrospective study was conducted on 30 uncontrolled diabetic patients (10 men and 20 women, mean age = 55.16 ± 2.2 years), 30 controlled diabetic patients (13 men and 17 women, mean age = 50.76 ± 1.97 years), and 30 healthy individuals who were companions to patients or came for their annual check-ups (14 men and 16 women, mean age = 49 ± 1.4 years) and referred to the Diabetes Center of Shahid Bahonar Hospital, Kerman University of Medical Sciences, Kerman, Iran from July to December, 2016.

The inclusion criteria were as follows: age above 30 years-old, fasting for at least 8 hours before blood and saliva sampling. Unwilling participants, subjects suffering from severe diabetic complications or systemic diseases, smokers, cases of alcohol dependence and those who had received medications of other diseases for at least 4 weeks before study were excluded. According to the clinical data, participants were divided into three groups as follows: 1) uncontrolled diabetic (*n* = 30); 2) controlled diabetic (*n* = 30); and 3) non-diabetic subjects as a control group (*n* = 30).

Participants with one of the following criteria were diagnosed as T2DM: 1) Fasting blood sugar (FBS) levels over 126 mg/dl; 2) random blood glucose levels over 200 mg/dl; 3) two-hour blood sugar levels over 120 mg/dl; A random plasma glucose (PG) measurement of ≥200 mg/dL met the criteria for a diagnosis of T2DM in patients with classic symptoms of hyperglycaemic crisis (increased thirst, Blurred vision, Frequent urination, Increased hunger).

Numbness or tingling in the feet. Furthermore, patients receiving antidiabetic medication with HbA1C ≥6.5% were labeled as uncontrolled T2DM, while those with diabetes criteria and HbA1c ≤6.5% were considered as controlled T2DM [24]. Written informed consents were obtained from all participants. This study conforms to the Declaration of Helsinki [25] regarding research involving human subjects and approved by the ethics committee of Kerman University of Medical Sciences (IR.KMU.REC.1395.364).

Frequent abscesses, lesions and oral mucosa abnormalities including oral candidiasis manifestations (white plaque, erythematous candidiasis, thrush, angular cheilitis, median rhomboid glossitis and denture stomatitis) were evaluated in all participants with clinical diagnosis of calibrated examiner. The location of any noted lesions were recorded [8]. The number of decayed, missing and filled teeth (DMFT) was recorded using the World Health Organization (WHO) recommendations for assessing oral health [26]. In addition, periodontal status was evaluated using the periodontal disease index (PDI) as follows: three components of each six selected teeth (upper left central incisor and first premolar, first upper right molar, lower left first molar and lower right central incisor and first premolar) were evaluated separately. All participants were evaluated on tongue blade sign. Xerostomia was characterized using Fox et al. questionnaire [27].

First, participants were asked to rinse their mouth 3 times with filtered water. Then, unstimulated saliva samples were collected in the morning between 8 and 11 a.m. after 5 min of rest by spitting out to a sterile glass tubes. The samples were immediately taken to a laboratory and centrifuged (at 3000 rpm for 15 min). Then, supernatants were immediately frozen and stored at − 20 °C. Salivary IgA concentration was measured in all participants by a commercially available enzyme-linked immunosorbent assay (ELISA) kit (Dia Metra, Milano, Italy). Experiments were carried out according to the manufacturer’s protocol. Beside, salivary levels of amylase were analyzed using an automatic analyzer (TechniCon Systems Inc., California, USA). Demographic and clinical information was compiled using questionnaires.

### Statistical analysis

Numerical variables were presented as mean ± SEM (standard error of mean), while categorical variables were summarized as numbers (percentages). The Kolmogorov-Smirnov test was used to evaluate the distribution of quantitative variables. The variables were compared between two groups by Student’s t-test and the Chi-square/Fisher’s Exact Test. Salivary levels of amylase and IgA were compared using one-way ANOVA with post-hoc Tukey multiple comparisons test across the three studied groups. Association between quantitative variables was assessed using *Pearson’s* correlation coefficient (r). The statistical analyses were performed using the SPSS software version 18.0 for Windows (SPSS Inc., Chicago, IL). *P*-values <0.05 were considered statistically significant.

## Results

**Fig. 1:**
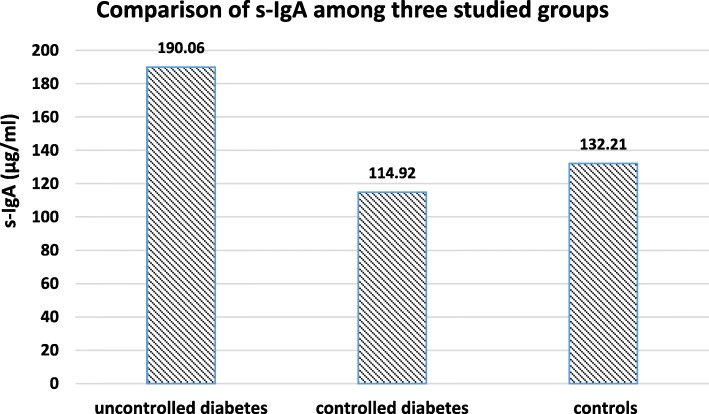
Comparision of s-IgA among three studied groups. Significant diffrence was seen between controlled diabetecs and uncontrolled diabetecs

The mean age of participants was 55.16 ± 2.2, 50.77 ± 1.7, and 49 ± 1.4 in uncontrolled diabetic, controlled diabetic and control group, respectively. There were no significant differences in age and sex among three groups (P>0.05 for both). Comparison of s-IgA among three studied groups are shown in Fig. 1. A significant difference of s-IgA level was found among three groups (*P* ≤ 0.0001). There was a significant higher level of s-IgA in uncontrolled diabetic patients compared to controlled diabetics (*P* ≤ 0.0001) and control subjects (*P* = 0.004). However, no significant difference was found between controlled diabetic and control subjects (*P* = 0.583).

**Fig. 2:**
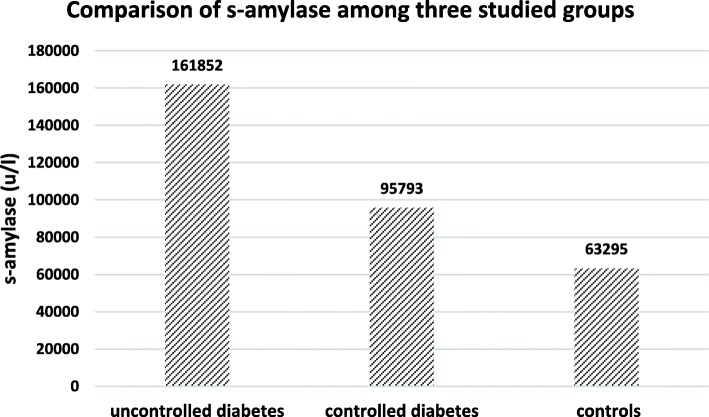
Comparison of s-amylase among three studied groups. Significant diffrence was seen between controlled diabetecs and uncontrolled diabetecs

Figure 2 shows the mean levels of s-amylase among three groups. There was a significant difference of s-amylase among three groups (P ≤ 0.0001). The mean levels of s-amylase in uncontrolled patients showed significant differences compared to controlled diabetic (*P* = 0.01) and control subjects (P ≤ 0.0001). However, the mean levels of s-amylase did not show significant differences between controlled diabetic and control subjects (*P* = 0.308).

Comparison of the mean levels of s-IgA and s-amylase with oral/dental manifestations among three studied groups are presented in Table 1. The mean levels of s-IgA and s-amylase in subjects with oral candidiasis were significantly different among three groups (*P* = 0.002 and *P* = 0.018, respectively). The results of post-hoc Tukey test illustrated that the mean levels of s-IgA in uncontrolled diabetic patients with oral candidiasis (195.79 ± 17.78) were significantly higher than controlled diabetic patients (98.13 ± 10.67) (*P* = 0.003). Similarly, the mean levels of s-amylase in uncontrolled diabetic patients with oral candidiasis (193,364.67 ± 36,135.98) were significantly higher than controlled diabetic patients (48,719.37 ± 28,372) (*P* = 0.028). Uncontrolled diabetic patients (210.76 ± 13.12) with white plaques had significantly higher level of s-IgA compared to controlled diabetics (81.83 ± 22.68) (*P* = 0.013). The results revealed that controlled diabetic patients (216,400 ± 23,950.4) had significantly lower levels of s-amylase concentration compared to the uncontrolled (25,150 ± 2361.5) ones (*P* = 0.038) There was a significant difference in the mean levels of s-IgA between uncontrolled (221.98 ± 39.76) and controlled diabetic patients (77.84 ± 23.19) with abscesses (*P* = 0.022). Moreover, the mean levels of s-IgA in uncontrolled patients (205.7 ± 20.87) with xerostomia were significantly higher than those of controlled diabetics (81.75 ± 12.1) (*P* ≤ 0.0001). Furthermore, no significant difference was found in s-IgA and s-amylase levels of subjects with trush or median rhomboid glossitis signs among three groups (P>0.05). Since angular cheilitis manifestation was not observed in participants, hence, it was not investigated in the analyses.

**Table 1:** Comparison of the mean levels of s-IgA and s-amylase with oral/dental manifestations among the three studied groups

Oral/dental manifestations	s-IgA				s-Amylase			
	uncontrolled diabetic	controlled diabetic	control	*p*-value	uncontrolled diabetic	controlled diabetic	control	*p*-value
Oral candidiasis	195.79 ± 17.78	98.13 ± 10.67*	107.52 ± 32.69	0.002	193,364.67 ± 36,135.98	48,719.37 ± 28372*	42,078.33 ± 30,461.78	0.018
White plaque	210.76 ± 13.12	81.83 ± 22.68	–	0.013	307,490 ± 175,903.1	17,760.5 ± 1239.5	–	0.292
Erythematous candidiasis	209.88 ± 49.64	110.26 ± 23.2	–	0.436	216,400 ± 23,950.4	25,150 ± 2361.5	–	0.038
Thrush	221.85 ± 33.63	73.35 ± 6.73*	172.85 ± 8.3	0.57	136,500 ± 26,513.61	138,037 ± 108,821	12,035 ± 8651	0.254
Median rhomboid glossitis	163.13 ± 50.12	121.48 ± 16.93	74.85 ± 2.24	0.215	93,200 ± 37,900	17,670 ± 3452.91	57,100 ± 18,338.8	0.257
abscesses	221.98 ± 39.76	77.84 ± 23.19	–	0.022	284,466.66 ± 76,543.8	100,500 ± 53,700	0.243	0.243
Xerostomia	205.7 ± 20.87	81.75 ± 12.1*	118.49 ± 33.96	0.0001	189,807.6 ± 44,143.3	59,417.2 ± 2463.08	77,973.3 ± 26,897.8	0.065

s-IgA, salivary immunoglobulin A; s-amylase, salivary amylase. * Significant difference with uncontrolled diabetes (*P*<0.05). Significant at <0.05 levels

Correlations among age, FBS, HbA1C, DMFT (Decayed, Missing, Filled Teeth), PDI (Periodontal Disease Index), s-IgA, and s-amylase within controlled diabetic patients are illustrated in Table 2. Results show that there is a positive correlation between age and DMFT and age and PDI (r = 0.703, *P* ≤ 0.0001 and r = 0.504, *P* = 0.005, respectively). Also, a significant correlation was observed between DMFT and PDI (r = 0.694, *P* ≤ 0.0001). However, other correlations were too poor to affect each other.

**Table 2:** Correlations among age, FBS, HbA1C, DMFT, PDI, s-IgA, and s-amylase within controlled diabetic patients

		Age	FBS	HbA1C	DMFT	PDI	s-IgA	s-Amylase
Age	Pearson’s Correlation	1.000	−0.294	− 0.189	0.703	0.504	0.324	−0.144
	*P*-value	–	0.115	0.316	.000	.005	0.081	0.449
FBS	Pearson’s Correlation	−0.294	1.000	0.429	−0.278	− 0.154	− 0.057	− 0.222
	*P*-value	0.115	–	0.018	0.137	0.415	0.766	0.239
HbA1C	Pearson’s Correlation	−0.189	0.429	1.000	− 0.135	0.064	− 0.141	− 0.166
	*P*-value	0.316	0.018	–	0.478	0.736	0.457	0.381
DMFT	Pearson’s Correlation	0.703	−0.278	−0.135	1.000	0.694	−0.41	−0.112
	*P*-value	.000	0.137	0.478	–	.000	0.83	0.555
PDI	Pearson’s Correlation	0.504	−0.154	0.064	0.694	1.000	−0.127	−0.159
	*P*-value	.005	0.415	0.736	.000	–	0.503	0.403
s-IgA	Pearson’s Correlation	0.324	−0.057	−0.141	−0.41	− 0.127	1.000	− 0.022
	*P*-value	0.081	0.766	0.457	0.83	0.503	–	0.909
s-Amylase	Pearson’s Correlation	−0.144	−0.222	0.166	−0.112	−0.159	− 0.022	1.000
	*P*-value	0.449	0.239	0.381	0.555	0.403	0.909	–

s-IgA, salivary immunoglobulin A; s-amylase, salivary amylase. Significant at <0.05 levels

Table 3 shows the correlations among age, FBS, HbA1C, DMFT, PDI, s-IgA, and s-amylase within uncontrolled diabetic patients. Significant positive correlations were found between age and FBS, DMFT, and PDI (*r* = 0.393, *P* = 0.031, *r* = 0.395, *P* = 0.031, and *r* = 0.536, *P* = 0.002, respectively). In addition, a significant positive correlation was seen between PDI and DMFT (*r* = 0.922, *P* ≤ 0.0001). However, other correlations were not significantly different.

**Table 3:** Correlations among age, FBS, HbA1C, DMFT, PDI, s-IgA, and s-amylase within uncontrolled diabetic patients

		Age	FBS	HbA1C	DMFT	PDI	S-IgA	S-Amylase
Age	Pearson’s Correlation	1.000	0.393	0.152	0.395	0.536	0.018	−0.271
	*P*-value	–	0.031	0.423	0.031	0.002	0.923	0.148
FBS	Pearson’s Correlation	0.393	1.000	0.015	0.067	0.058	−0.181	−0.293
	*P*-value	0.031	–	0.939	0.723	0.762	0.338	0.116
HbA1C	Pearson’s Correlation	0.152	0.015	1.000	−0.037	0.031	0.039	0.172
	*P*-value	0.423	0.939	–	0.846	0.873	0.837	0.362
DMFT	Pearson’s Correlation	0.395	0.067	−0.037	1.000	0.922	−0.061	− 0.321
	*P*-value	0.031	0.723	0.846	–	0.000	0.749	0.084
PDI	Pearson’s Correlation	0.536	0.058	0.031	0.922	1.000	−0.017	−0.256
	*P*-value	0.002	0.762	0.873	0.000	–	0.927	0.172
s-IgA	Pearson’s Correlation	0.018	−0.181	0.039	−0.061	− 0.017	1.000	0.147
	*P*-value	0.923	0.338	0.837	0.749	0.927	–	0.438
s-Amylase	Pearson’s Correlation	−0.271	− 0.293	0.172	− 0.321	− 0.256	0.147	1.000
	*P*-value	0.148	0.116	0.362	0.084	0.172	0.438	–

s-IgA, salivary immunoglobulin A; s-amylase, salivary amylase. Significant at <0.05 levels

Table 4 illustrates the correlations among age, FBS, HbA1C, DMFT, PDI, s-IgA, and s-amylase within healthy subjects. Similar to two previous groups, a significant positive correlation was observed between age and DMFT and age and PDI in the control group (r = 0.641, P ≤ 0.0001 and r = 0.514, *P* = 0.004, respectively). Also, a significant positive correlation between DMFT and PDI was observed. A significant positive correlation was found between FBS and HbA1C (r = 0.669, P ≤ 0.0001). Moreover, significant positive correlations were found between s-IgA and DMFT and s-IgA and PDI (r = 0.444, *P* = 0.014 and r = 0.386, *P* = 0.035, respectively). However, no significant correlation was observed among other variables in the control group.

**Table 4:** Correlations among age, FBS, HbA1C, DMFT, PDI, s-IgA, and s-amylase within healthy subjects

		Age	FBS	HbA1C	DMFT	PDI	s-IgA	s-Amylase
Age	Pearson’s Correlation	1.000	− 0.135	−0.255	0.641	0.514	0.307	− 0.142
	*P*-value	–	0.478	0.174	0.000	0.004	0.099	0.455
FBS	Pearson’s Correlation	−0.135	1.000	0.669	0.011	−0.039	0.05	0.096
	*P*-value	0.478	–	0.000	0.953	0.836	0.791	0.613
HbA1C	Pearson’s Correlation	−0.255	0.669	1.000	−0.108	− 0.258	− 0.055	− 0.096
	*P*-value	0.174	0.000	–	0.570	0.168	0.774	0.613
DMFT	Pearson’s Correlation	0.641	0.011	−0.108	1.000	0.720	0.444	−0.125
	*P*-value	0.000	0.953	0.570	–	0.000	0.014	0.512
PDI	Pearson’s Correlation	0.514	−0.039	− 0.258	0.720	1.000	0.386	−0.127
	*P*-value	0.004	0.836	0.168	0.000	–	0.035	0.504
s-IgA	Pearson’s Correlation	0.307	0.05	−0.055	0.444	0.386	1.000	0.203
	*P*-value	0.099	0.791	0.774	0.014	0.035	–	0.281
s-Amylase	Pearson’s Correlation	−0.142	0.096	−0.096	− 0.125	−0.127	0.203	1.000
	*P*-value	0.455	0.613	0.613	0.512	0.504	0.281	–

s-IgA, salivary immunoglobulin A; s-amylase, salivary amylase. Significant at <0.05 levels

## Discussion

Findings of the present study demonstrated that salivary IgA and amylase levels are associated with diabetes. Moreover, s-IgA levels are associated with T2DM in patients with oral candidiasis, white plaque, abscesses, or xerostomia manifestations, while s-amylase levels are associated with T2DM in patients with oral candidiasis or erythematous candidiasis manifestations.

Alterations in oral/dental manifestations and salivary secretions have been proposed in the development, symptomatology, and severity of some systemic diseases such as diabetes [1]. Various studies have evaluated alterations in salivary amylase and IgA levels in diabetic patients and have yielded conflicting results [5, 8, 13, 20, 28]. To the best of the authors’ knowledge, no study has yet investigated the association of salivary levels of Ig-A and amylase with oral/dental manifestations in patients with uncontrolled and controlled T2DM. Therefore, this study measured unstimulated saliva to evaluate IgA and amylase levels and their associations with oral/dental manifestations among 30 patients with uncontrolled T2DM, 30 patients with controlled T2DM, and 30 control subjects.

In the present study, significantly higher levels of s-IgA were observed in the uncontrolled diabetic patients compared to the controlled diabetic and the healthy subjects. It is plausible that increased mean levels of s-IgA are caused by the presence of plaque, calculus, and infection in the diabetic patients in this study. These findings were in agreement with those of previous studies [27, 29–33]. Conversely, Salles et al. [34] reported a lower s-IgA concentration in diabetic patients compared to non-diabetic individuals, which is probably related to the non-homogeneous gender distribution and the use of both T1DM and T2DM patients in their research. Another study conducted by Bhuyan et al. [35] demonstrated lower level of s-IgA in diabetic patient’s specially uncontrolled diabetic ones. Moreover, others found no significant differences [5, 28, 36]. However, none of them compared the s-IgA among controlled T2DM, uncontrolled T2DM and healthy subjects separately. These discrepancies in findings may be explained by variations in saliva collection methods (stimulated vs. unstimulated), disease stages, genetic susceptibility, or study design and measuring methods, e.g., the inclusion of three groups (uncontrolled T2DM, controlled T2DM, and healthy participants) vs. two groups (diabetic and non-diabetic participants) as study populations, or the use of ELISA method to measure s-IgA vs. immunoturbidimetric or immunonephelometric methods.

The current findings showed no significant difference of s-amylase levels between the controlled diabetic group and the healthy control group. However, a significantly higher concentration of s-amylase in the uncontrolled diabetic group was observed compared to other two groups. This finding supports the previous studies [18–20, 37] . While Yavuzimaz et al. [32] and Prathibha et al. [17] reported significant decreases in s-amylase levels in controlled diabetic patients when compared with healthy subjects. However, other studies showed no significant difference which was not consistent with the current results [13, 23, 38]. It is postulated that increased permeability of salivary glands basement membranes leads to the higher levels of s-amylase in diabetic groups. These discrepancies could be attributed to the differences in genetic susceptibility, case matching and measuring techniques. Therefore, further research is required to clarify the exact mechanisms.

Previous studies have been investigated the association of salivary components, such as Ig-A, with different oral conditions [3, 6, 8, 35]. In the current study, uncontrolled diabetic patients with oral candidiasis, white plaque, abscesses, or xerostomia had higher s-IgA levels compared to the controlled diabetic participants; however, no significant differences were observed between the uncontrolled diabetic patients and the healthy participants. A leading mechanism proposed to explain the increased levels of s-IgA which may be due to the fact that T2DM affects the stimulation of immune response and secretion of s-IgA as a result of oral-dental disturbance. Our previous study showed higher levels of s-IgA in diabetic patients [8], while s-IgA was significantly lower in diabetic patients with oral candidiasis compared to healthy subjects and no significant differences were found among other oral-dental manifestations between two groups.

The salivary enzyme alpha-amylase mainly acts in the digestion of carbohydrates. It also has an important role in mucosal immunity by inhibiting the bacteria function [39]. In the current study, uncontrolled diabetic patients with oral candidiasis or erythematous candidiasis showed significant higher levels of s-amylase compared to controlled diabetic patients with the mentioned oral manifestations, however, no significant differences were found between uncontrolled diabetic patients and healthy subjects. The increased levels of s-amylase in uncontrolled diabetic patients may be explained by the increase of oral-dental manifestations to inhibit the microorganism function [40–42].

Previous studies documented that the risk of destructive periodontitis and other oral-dental manifestations is increased in T2DM [43, 44]. In agreement with other reports [43–45], the present study demonstrated that both patients with uncontrolled and controlled T2DM had poorer health in some oral-dental conditions compared to the control group subjects (results are not shown). Thus, more comprehensive and regular oral-dental assessments at an early stage would be imperative in these patients.

An impressive body of evidence supports the concept that oral-dental conditions are generally worsened by increasing age [46–48]. As expected, a significant positive correlation was observed between age and DMFT and age and PDI in each of the three studied groups; this result is compatible with those of previous studies [8, 49]. Towards this end, a significant positive correlation was found between DMFT and PDI in each three studied groups. Furthermore, significant positive correlations were observed between s-IgA and DMFT and s-IgA and PDI in healthy subjects; this result was in accordance with our previous study which had showed that s-IgA had been positively correlated with PDI [8]. No significant correlation was seen between s-IgA and the other variables in the uncontrolled and controlled diabetic groups; this result was in contrast with those of the authors’ previous study which had showed positive correlations between s-IgA and HbA1C, DMFT, and PDI in diabetic patients [8]. It is noteworthy that no significant correlation was found between s-amylase and other variables in the three studied groups. Given the few studies regarding the correlation of s-amylase and other factors, further studies are highly recommended to assess these correlations.

The cost-effectiveness, accessibility, and non-invasive characteristics of saliva samples are the most important advantages of using this body fluid for screening and diagnosing various metabolic diseases [5, 50]. Moreover, previous studies documented that saliva fluid is an accurate and reliable tool for comparing blood measurements [1, 5].

It is well known that genetic background has an important impact on s-IgA and s-amylase levels. Moreover, individual diet can directly affect s-IgA and s-amylase levels. Likewise, the secretion and synthesis of s-IgA and s-amylase are strongly regulated by the neuroendocrine system; hence, neuroendocrine-related factors such as stress, physical activity, psychosocial conditions, and menstrual cycle may affect s-IgA and s-amylase levels [49, 51, 52]. Nevertheless, further longitudinal studies with a larger population are highly recommended to support the findings of the present study.

## Conclusion

Higher s-amylase and s-IgA concentrations may reflect oral-dental manifestations in T2DM. Moreover, the current findings suggest that s-amylase and s-IgA may serve as a complementary and alternative fluid in screening diabetes mellitus.

## Data Availability

The datasets used and analyzed during the current study are available from the corresponding author on reasonable request.

## Abbreviations

DMFT: number of decayed, missing and filled teeth
ELISA: enzyme-linked immunosorbent assay
FBS: Fasting blood sugar
HbA1C: hemoglobin A1c
PDI: periodontal disease index
PG: plasma glucose
S-amylase: salivary amylase
S-IgA: salivary immunoglobulin-A
T2DM: Type 2 diabetes mellitus
WHO: World Health Organization
